# Factors Related to Presenteeism among South Korean Workers Exposed to Workplace Psychological Adverse Social Behavior

**DOI:** 10.3390/ijerph17103472

**Published:** 2020-05-15

**Authors:** Jee-Seon Yi, Hyeoneui Kim

**Affiliations:** School of Nursing, Duke University, Durham, NC 27710, USA

**Keywords:** presenteeism, adverse social behavior, work, Korean Working Conditions Survey, South Korea

## Abstract

Presenteeism negatively affects both individuals and society. This study identified factors of presenteeism among workers in South Korea, especially in relation to exposure to adverse social behaviors. Here, an adverse social behavior refers to any forms of workplace violence or intimidation. This study used the data from 23,164 full-time salaried employees, who participated in the fifth Korean Working Conditions Survey. This study attempted to predict presenteeism based on the exposure to adverse social behaviors and working conditions using logistic regression. Presenteeism was reported in 15.9% of the sample. Presenteeism was significantly higher among workers with the following characteristics: females, aged 40 years or older; middle school graduates; over 40 working hours a week; shift workers; no job-related safety information received; exposure to adverse social behavior and discrimination; and those with a high demand for quantitative work, low job autonomy, high emotional demands, and high job stress. The workers exposed to adverse social behavior showed a higher prevalence of presenteeism (41.2%), and low job autonomy was the most significant predictor of presenteeism. The findings of this study suggest that allowing enough autonomy in job-related roles may help alleviate presenteeism among those who have experienced adverse social behavior at work.

## 1. Introduction

Presenteeism has been used to refer to two slightly different meanings: people going to work sick [1,2,3,4], or more specifically, the ranges of negative impact on productivity brought by workers not fully functioning at the workplace due to an illness [5]. Currently, the former is more widely adopted [6]. Presenteeism negatively affects both individuals and society [7] by forcing people to go to work despite feeling sick [1,2,3,4], which results in decreased productivity and further aggravation of health [5]. Presenteeism increases the risk of health problems with future episodes of sickness absence [3,7,8,9], reduces workers’ efficiency/productivity, and leads to greater losses to the employers than sickness absences [10,11,12]. Although presenteeism is an important global phenomenon [2,9,13], it has been less actively studied compared to absenteeism or sick leave [14,15].

### 1.1. Related Theories

According to several theories of presenteeism, the determinants of presenteeism are mostly related to work, individual health, and stress [16]. Presenteeism has been reported to have a greater association with work-related factors than absenteeism [17]. Psychological factors, such as emotional fatigue and negative strain, have been reported to be positively related to presenteeism [18]. The job demands-resources model explains psychological factors related to the working environment; presenteeism occurs because high job demands cause exhaustion, but workers cannot leave work due to limited job resources [19]. Several studies have attempted to explain presenteeism with psychological factors, and those reported that psychological factors were a significant impact on presenteeism. However, research on which psychological factors impact on presenteeism is still insufficient [20].

Presenteeism should be connected with the working environment because it is affected by an institution [20]. Pohling et al. [21] explained presenteeism in terms of occupational environmental factors based on the theory of person–environment fit, especially mismatch between the person and the job, arguing that employees experience work-related stress when the actual experience at the workplace is mismatched to their needs or expected working conditions. Person–job mismatch can cause psychological burnout among workers. However, in terms of resource conservation theory, workers go to work even when their health is poor because, although their performance or ability does not meet their work demands, there is a possibility of a significant loss of resources otherwise [18].

Based on these theories, previous studies have reported specific characteristics of the workers who decide on presenteeism. However, as most studies assumed that workers are in a healthy state without exposure to hazards, they could not show the specific factors of workers who have already been exposed to risk factors in the workplace that lead them to presenteeism.

To develop and communicate suitable interventions that can help deal with this phenomenon study on presenteeism should consider the context in which the behavior occurs [20]. In addition, different health statuses require different adjustments because the subjects’ demands differ with their restrictions [22]. Therefore, it is necessary to identify the factors that cause workers to decide on presenteeism, even though they are already exposed to psychological risk factors at work. This can also provide evidence for more practical management methods regarding what to manage if there has already been exposure to risk at work. An unsafe workplace is one of the psychological risk factors in the workplace [23]. Recently, psychological violence and harassment have become more common threats to most workers than physical violence [24].

Presenteeism has differences between nations [20]. According to the research framework for the content of a decision-integrated presenteeism model, presenteeism is affected by the factors related to the person, work, and organization, all of which are also affected by the environmental influences such as culture and society [2]. This framework effectively explains Korean workplace culture, where going to work and staying at work for long hours, even if a person is not feeling well, is considered desirable [25]. This behavior can be attributed to groupism, patriarchal tendencies, and an ingrained sense of diligence [25].

### 1.2. Presenteeism and Adverse Social Behavior

All acts of violence and intimidation at work, such as bullying, harassment, and violence in the workplace, are defined as an adverse social behavior at workplace [26]. Workers exposed to an adverse social behavior feel overburdened due to always being worried about their safety, which has numerous ramifications for one’s health (e.g., doubling the risk of harming one’s mental health) [27], and can lead to presenteeism [28]. Indeed, a prior study reported that exposure to psychosocial risk factors can increase the risk of presenteeism [9]. McGregor et al. found that psychological job demands, including adverse social behavior in the workplace, were related to presenteeism [29].

Therefore, examining how the experience of adverse social behaviors in the workplace, a subtle and non-physical factor, affects presenteeism is an area much needing research. In particular, it is necessary to identify the factors for presenteeism that apply only in workers who are exposed to psychologically adverse social behaviors. However, previous research on presenteeism has mostly included workers as a single group or did not consider adverse social behaviors as an antecedent risk that threatens the health of the subject, including only working conditions. Instead, they have mostly focused on the health problems, reduced productivity, and specific job types of workers [30].

### 1.3. Study Aims

Studying presenteeism is particularly important in the context of the South Korean workplace, where overwork leads to many social problems including employee suicide. Examining presenteeism may elucidate the causes of many workplace related issues, and also, identify potential solutions for them. Nonetheless, there are few studies on presenteeism in South Korea [25].

By analyzing national workers survey data from South Korea, this study aimed to inform the development of workplace policies and managerial strategies that effectively address presenteeism among South Korean workers exposed to psychologically adverse social behavior. The specific aims were: (a) to identify the factors of presenteeism among workers in general, and (b) to identify the related factors of presenteeism among those who have been exposed to adverse social behavior.

## 2. Methods

This study was a secondary analysis of the data collected through the fifth Korean Working Conditions Survey of the Occupational Safety and Health Research Institute (OSHARI) in South Korea. It was approved by the Institutional Review Board (IRB) of D University (Pro00104026).

### 2.1. Setting and Participants

This study used the data from 23,164 full-time salaried workers among 50,205 employees who participated in the fifth Korean Working Conditions Survey (KWCS). The Korean Working Conditions Survey is a government-approved survey that has been conducted since 2006 to identify work-related risk factors among South Korean employees. The survey questionnaires were developed by researchers and government administrators in the field of occupational safety and health, benchmarking the European Working Conditions Survey (EWCS) and the UK’s Labour Force Survey (LFS). The initial set of questions were validated through a face-to-face interview of workers to ensure the correct understanding of the question. The questionnaire was finalized, incorporating the input from Statistics Korea and the National Institute of Korean Language [31].

This survey targets all employees in South Korea who are economically active, older than 15 years old, and worked for more than 1 h for income purposes over the previous week at the time of the survey. The participants were selected from across South Korea, using a multi-stage systematic cluster sampling. Trained interviewers visited the selected homes and gathered the data using the computer-assisted personal interviewing (CAPI) method. The datasets generated by this survey series have been used in numerous occupational health studies in South Korea [32,33,34].

### 2.2. Measurements

#### 2.2.1. Demographic Characteristics

Demographic variables included for this study are gender, age, education level, and income.

#### 2.2.2. Occupation Characteristics

The job-related information incorporated in the analysis are job type, hours worked per week, company size, years of service, work shift, and safety information provided at work.

#### 2.2.3. Adverse Social Behavior

The respondents were asked to answer “yes” or “no” regarding whether they had ever been exposed to various adverse social behaviors, such as verbal abuse, unwanted sexual attention, threats, or humiliating behavior from clients at work, over the past one month. This study considered that the respondents were exposed to an adverse social behavior if they answered “yes” to one or more of these items.

#### 2.2.4. Discrimination

The participants were asked whether they had experienced discrimination at work during the past 12 months in relation to age, ethnic group, nationality, gender, religion, disability, sexual orientation, academic background, hometown, and employment type. If the participants answered “yes” to at least one item, they were deemed to have experienced discrimination.

#### 2.2.5. Working Conditions

Working conditions consisted of quantitative and emotional demands at work, job autonomy, job stress, and social support from colleagues and managers. Each variable was classified based on the criteria provided in the sixth EWCS [26].

The question on quantitative demands inquired if the respondents had “enough time to do their jobs”, and answers of “never” or “rarely” were assumed to indicate a high degree of quantitative demands, and “always”, “most of the time” or “sometimes” were assumed to indicate a low degree of quantitative demands. Job autonomy was determined via the interdependency of five items that can affect the completion of work, such as: (1) the work done by colleagues, (2) direct demands from people, such as clients, passengers, pupils, patients, (3) numerical production targets or performance targets, (4) the automatic speed of a machine or movement of a product, (5) the direct control of their boss. If the respondents selected three or more items “yes”, they were considered to have a low degree of job autonomy. As for emotional demands, the participants were asked if they were hiding their feelings at work. If the answers of “most of the time” or “always” were considered as high emotional demands and “never”, “rarely” or “sometimes” were considered as low emotional demands.

Different levels of job stress were defined based on the responses to the item, “I am under stress at work”. If the respondents indicated “always”, “most of the time” or “sometimes”, they were considered experiencing a high degree of stress and “never”, “rarely” or “sometimes” were considered to be experiencing a low degree of stress. Regarding social support from colleagues and managers, the respondents were asked how often they receive “help/support from colleagues” and “help/support from managers”. If they marked “most of the time” or “always”, they were deemed to have a high degree of social support, and “never”, “rarely” or “sometimes” were deemed to have a low degree of social support.

#### 2.2.6. Presenteeism

To discern presenteeism, the participants were asked, “Over the past 12 months, have you ever come to work even though you were sick?” [35]. If they answered “yes”, they were regarded to have experienced presenteeism.

### 2.3. Statistical Analysis

Data analysis was performed using SPSS 25.0, with a 95% significance level. Frequencies, percentages, and chi-square tests were obtained to describe the characteristics and presenteeism of the participants. Using complex samples of logistic regression, the study attempted to predict presenteeism based on the respondents’ demographics, occupational characteristics, exposure to adverse social behavior, discrimination, and working conditions.

This analysis used a weighted sample to increase the estimates’ accuracy by matching the composition of the population, thus, preventing sampling bias. The weights were used by multiplying the sampling weight, the non-response weight, and the post-stratification weight.

## 3. Results

### 3.1. Characteristics and Presenteeism among the Participants

Table 1 displays the characteristics of the 23,164 workers included in the analysis. The sample included more males (59.6%) and those with age of 40 or older (54.9%). The majority of the sample had at least college level of education (67.9%) and reported no experience of adverse social behavior (95.6%), discrimination (85.9%), or presenteeism (84.1%). The majority of the sample experienced high job emotional demands (80.6%) and high job-related stress (83.7%). Social support at the workplace and job autonomy were also reported to be high.

When compared by the exposure to an adverse social behavior, similar distributions of the sample characteristics were observed in the subgroups; the sample characteristics are significantly different except for age, company size, social support from colleagues and managers. The exposure group included more service and sales workers (43.9%) and reported a slightly higher rate of discrimination, quantitative demands, emotional demands, and job stress. The exposure group reported a higher rate of presenteeism (41.2%).

### 3.2. Factors Related to Presenteeism

Table 2 shows how the above-mentioned factors were associated with presenteeism. Presenteeism was significantly higher among females (OR = 1.606, 95% CI = 1.436–1.797) and those aged 40 years or older (OR = 1.209, 95% CI = 1.081–1.353). A lower level of education attainment (middle school or lower) led to higher presenteeism than college or higher level of education (OR = 1.416, 95% CI = 1.122–1.786). Extended working hours were also associated with a higher level of presenteeism. Other factors associated with a significantly higher level of presenteeism were shift workers (OR = 1.277, 95% CI = 1.101–1.428), those who did not receive safety information at work (OR = 1.133, 95% CI = 1.020–1.258), those with exposure to adverse social behavior (OR = 2.965, 95% CI = 2.452–3.585), and those who have experienced discrimination (OR = 1.607, 95% CI = 1.412–1.829). In terms of working conditions, presenteeism was higher among those with a high quantitative demand (OR = 1.636, 95% CI = 1.440–1.858), low job autonomy (OR = 1.588, 95% CI = 1.407–1.793), high emotional demands (OR = 1.392, 95% CI = 1.204–1.611), and high job stress (OR = 1.543, 95% CI = 1.315–1.812). The income, high school education, job type, years of service, and company size did not show any significant effect on presenteeism.

### 3.3. Presenteeism among Workers Exposed to Adverse Social Behavior

Table 3 depicts the factors associated with presenteeism among workers who have been exposed to an adverse social behavior. In a separate analysis of this group, only job autonomy (OR = 1.687, 95% CI = 1.102–2.580) remained as the factor that significantly affects presenteeism. A lower job autonomy was associated with a higher level of presenteeism. When analyzed separately, the sample with those who have not been exposed to adverse social behavior did not show notable differences from the entire sample in terms of the factors that significantly contributed to presenteeism except the job type (Table 4). Professional workers were more likely to experience presenteeism among those who have not experienced an adverse social behavior. The analysis of the entire sample also showed that professional workers were likely to have more presenteeism, but that trend was not statistically significant.

## 4. Discussion

While supporting the findings of previous studies that identified contributing factors of presenteeism, the results of this research extended the current understanding of presenteeism by providing additional understanding of the factors that potentially contribute to presenteeism among workers who have been exposed to adverse social behavior.

### 4.1. Factors Related to Presenteeism

This study demonstrated that the risk factors of presenteeism are multidimensional, ranging from organizational culture and job-related elements to individual differences. This finding is consistent with a previous study that showed that presenteeism is influenced by complex factors [36].

This analysis showed that women experience more presenteeism than men, which may be explained by the traits that females share to some extent. Previous research has reported that women tend to focus on others, display more symptoms of illness, and come to work even though they may be sick (which might also contribute to presenteeism) [18,37]. Those with lower levels of education are negatively affected both physically and mentally by workplace discrimination. Such a situation may contribute to the increased risk of presenteeism in the group [34].

Presenteeism is known to be more prevalent among younger workers [15]. However, the results of this study suggest that older workers (age ≥ 40 years) are more likely to exhibit presenteeism than their younger counterparts. Older workers may tend to “save up” time-off for “rainy days” such as when they are seriously ill or experience a family emergency. They may not think they need to stay home to recover from minor illnesses, and likely feel they should set an example for their colleagues [38,39].

The existing literature implies that a high degree of job demands, a high amount of pressure in terms of time, shift schedules, and long working hours are key risk factors of presenteeism [9,18,40,41]. Employees involved in such a highly demanding workplace (e.g., long working hours, shift schedules, fast-paced occupations, and a high amount of work) tend to feel pressured into showing up for work even when they are sick [34]. Similarly, people may feel that they have no choice but to report to work even if they feel unwell, when they have less authority to make decisions around their work, such as modifying schedules, and delegating tasks [9,19,42].

High job stress due to mental health or psychological problems is known as a major determinant of presenteeism [7,18]. Karasek’s job stress model explains job stress with job demands and workplace control [43]. This model argues that workers experience a high level of stress when they have a low degree of job autonomy, despite having high job demands over a long period of time [43]. Gilbreath and Karimi [4] also proved job stress and presenteeism were positively correlated, where negative behaviors of a supervisor had the strongest association with job stress-related presenteeism of employees. Hence, diverse job demands or restrictions encountered in one’s professional environment lead to psychological burdens and excessive mental tension, which, in turn, lead to presenteeism.

An expert panel convened by the American College of Occupational and Environmental Medicine (ACOEM) recommended considering emotional demands when designing measures to prevent presenteeism [44]. This study’s findings are also in line with the findings of a prior study that suggests emotional demands increase the risk of presenteeism [45].

Employees who are overworked or feel unsafe on the job are likely to become ill [22]. Therefore, a systematic intervention toward a safe working environment is necessary. This study revealed that exposure to adverse social behavior is the most vital predictor of presenteeism, which is supported by the existing literature [46]. This finding highlights the importance of treating adverse social behavior and discrimination as a critical safety threat that must be mitigated to ensure a safe working environment. Workers who have been exposed to adverse social conduct or discrimination feel more pressure to go to work as they fear being seen as disloyal, thus, deciding to go to work, even if they need to recuperate at home [47,48].

### 4.2. Presenteeism among Participants Exposed to Adverse Social Behavior

This study investigated the risk factors for presenteeism among workers who have been exposed to an adverse social behavior and revealed that job autonomy is the most significant predictor of presenteeism. This result supports previous research findings that if only unhealthy subjects are selected, the factors associated with presenteeism identified for healthy workers disappear or decrease in intensity [3]. Our results show that those with exposure to adverse social behavior experienced presenteeism at a rate of 41.2%. This rate is more than double that of all workers (15.9%), and clearly showed that nonphysical violence can cause a vulnerability toward presenteeism among victims.

Research has found that psychologically adverse social behavior is a significant risk factor for presenteeism [9]. However, it is challenging to prove its effects in a particular case, as the psychological injury inflicted is not easily visible. In addition, since people generally find it difficult to decide on the legitimacy of their sick leave without advice from experts [49], ironically, in some cases, they go to work to show that they truly are sick [22]. Therefore, it is important to recognize that workers exposed to psychologically adverse social behavior are vulnerable groups with high health risks and to manage them appropriately.

In particular, this study demonstrated that the level of job autonomy is a critical factor in presenteeism among workers exposed to adverse psychological social behavior that makes them more vulnerable to presenteeism [11,18,42,50]. In the job demand-control model, one’s ability to make choices is a crucial element that can alleviate the burden of the job [51]. Furthermore, this aspect can mitigate job stress from the employee’s perspective, even when there is an enormous amount of work.

However, managers often consider the degree of disease or stress to be at a level that workers can endure on their own and tend to take presenteeism positively. Furthermore, workers may think that their absence would be a burden on others by causing them to take over their work, and feel peer pressured to report to work even if they are sick (i.e., implicit principles of organization) [52,53]. Thus, the structures, policies, and normative characteristics of the working environment in which presenteeism occurs should be systematically reviewed in order that managers and executives recognize the issue as important and develop active institutional-level measures. In particular, such measures will have to block the psychological path leading to presenteeism by ensuring sufficient autonomy to workers who have been exposed to adverse psychological social behavior.

Presenteeism increases the risk of health issues and long-term absence [54]. However, while most OECD member nations support a paid sick leave system for individual diseases with laws and institutional regulations, the paid sick leave policies in South Korea mostly apply to work-related accidents or illnesses in most workplaces [55]. While some companies provide paid sick leave, the system is purposed for welfare, so institutional systems and cultures are important factors in determining presenteeism.

The employers in South Korea certainly need to revise their sick leave policies of their company [10,44] and need to consider covering non-work-related health issues of employees. Additionally, from a long-term standpoint, employers recognize presenteeism as a critical issue to resolve just as an employee illness or various managerial challenges [10,44] and develop a strategic plan for addressing presenteeism. Furthermore, companies should establish cultural norms that encourage workers to take time off to recuperate, create a supportive organizational culture, and foster a healthy working environment while ensuring sufficient worker autonomy.

### 4.3. Strengths and Limitations

This study has some limitations. First, the analysis was conducted using self-reported survey data, which represents subjective experiences. Therefore, the findings of this analysis may have limited generalizability. Second, because it is a cross-sectional study, the interpretation of the causality on the relevance of each factor to presenteeism should be made carefully. Third, as the study employs a secondary analysis of an existing dataset, the investigation of the presenteeism was limited to the factors available in the dataset. Fourth, this study did not conduct exhaustive analyses on the complex interaction effects that may exist among worker’s characteristics in relation to presenteeism. A follow up study will address this gap in the analysis.

Nevertheless, this study has several merits. First, it used the most comprehensive national survey on health and working conditions among South Korean workers. Second, it investigated the factors of presenteeism among employees exposed to adverse social behavior, a serious issue worldwide. Third, it broadened the understanding of presenteeism by suggesting directions to remedy workers’ circumstances and to establish a basis for practical measures.

## 5. Conclusions

Presenteeism is linked to diverse factors ranging from job-related ones to individual differences. This study showed that workers exposed to psychologically adverse social behaviors at work should be treated as an important target. Among those who have been exposed to an adverse social behavior, lack of job autonomy is the most critical risk factor of presenteeism. This study suggests that it is important to provide employees with sufficient autonomy for job-related roles in order to alleviate presenteeism among those who have been exposed to adverse social behavior.

Employers and companies may need to consider various strategies to effectively mitigate presenteeism by assessing whether employees are exposed to workplace adverse social behavior, and allowing workers exposed to workplace social behavior sufficient autonomy in conducting their work. In particular, we developed a specific strategic plan and objective standard for addressing presenteeism among workers exposed to psychologically adverse social behaviors. In addition, there is a need to recognize the exposure to adverse social behavior in the workplace as a serious health concern covered by sick-leave policies.

## Figures and Tables

**Table 1:** Characteristics and presenteeism among the participants.

			Total (*n* = 23,164)		Exposed to Adverse Social Behavior				
					No (*n* = 22,112)		Yes (*n* = 1052)		F^b^ (*p*)
			N (%)	F^a^ (*p*)	N (%)	F^a^ (*p*)	N (%)	F^a^ (*p*)	
Demographic	Gender	Male	11,818 (59.6)	576.960 ** (<0.001)	11,329 (59.9)	585.431 ** (<0.001)	489 (46.5)	3.319 (0.069)	10.770 ** (0.001)
		Female	11,346 (40.4)		10,783 (40.1)		563 (53.5)		
	Age	<40	9198 (45.1)	136.150 ** (<0.001)	8760 (45.1)	133.712 ** (<0.001)	438 (46.6)	2.944 (0.086)	0.611 (0.434)
		≥40	13,966 (54.9)		13,352 (54.9)		614 (53.4)		
	Education level	≤Middle school	1434 (4.4)	5435.609 ** (<0.001)	1357 (4.4)	5282.982 ** (<0.001)	77 (5.3)	169.306 ** (<0.001)	11.771 ** (<0.001)
		High school	7451 (27.7)		7040 (27.4)		411 (34.8)		
		≥College	14,269 (67.9)		13,705 (68.3)		564 (59.9)		
	Income (million won)	<1.00	540 (2.0)	3335.660 ** (<0.001)	518 (2.0)	3148.258 ** (<0.001)	22 (2.1)	202.475 ** (<0.001)	9.983 ** (<0.001)
		1.00–2.99	13,080 (53.1)		12,395 (52.6)		685 (63.1)		
		3.00–4.99	6744 (36.7)		6502 (37.1)		242 (29.8)		
		≥5.00	1349 (8.2)		1310 (8.4)		39 (4.9)		
Occupation	Job type	Professionals	4299 (22.2)	144.776 ** (<0.001)	4170 (22.5)	169.788 ** (<0.001)	129 (15.7)	41.044 ** (<0.001)	68.690 ** (<0.001)
		Clerks	6536 (32.1)		6375 (32.8)		161 (18.8)		
		Service and sales workers	6516 (21.4)		5972 (20.3)		544 (43.9)		
		Blue collar	5707 (24.3)		5489 (24.4)		218 (21.5)		
	Hours worked per week	≤40	13,003 (57.7)	2329.393 ** (<0.001)	12,578 (58.5)	7243.097 ** (<0.001)	425 (40.4)	8.706 ** (<0.001)	73.173 ** (<0.001)
		41–52	6833 (29.3)		6491 (29.2)		342 (32.3)		
		≥53	3288 (12.9)		3003 (12.3)		285 (27.3)		
	Company size	<50	16,678 (69.9)	4231.448 ** (<0.001)	15,897 (69.8)	4025.751 ** (<0.001)	781 (72.4)	206.093 ** (<0.001)	0.990 (0.371)
		50–249	2162 (11.2)		3651 (19.0)		159 (16.9)		
		≥250	3810 (18.9)		2075 (11.2)		87 (10.7)		
	Year of Service	<5	10,022 (42.2)	296.124 ** (<0.001)	9523 (42.0)	283.365 ** (<0.001)	499 (46.5)	32.383 ** (<0.001)	20.980 ** (<0.001)
		5–9	6002 (26.0)		5679 (25.7)		323 (32.9)		
		≥10	6663 (31.8)		6454 (32.4)		209 (20.7)		
	Work shift	No	20,402 (89.0)	9487.819 ** (<0.001)	19,654 (89.9)	9699.074 ** (<0.001)	748 (68.7)	87.461 ** (<0.001)	279.366 ** (<0.001)
		Yes	2754 (11.0)		2450 (10.1)		304 (31.3)		
	Safety information provided at work.	No	7156 (30.6)	2175.971 ** (<0.001)	6780 (30.4)	2132.229 ** (<0.001)	376 (36.1)	50.237 ** (<0.001)	9.305 * (0.002)
		Yes	15,893 (69.4)		15,230 (69.6)		663 (63.9)		
Adverse social behavior	No		22,112 (95.6)	12,396.751 ** (<0.001)					
	Yes		1052 (4.4)						
Discrimination	No		20,332 (85.9)	6829.564 ** (<0.001)	19,557 (86.5)	6700.086 ** (<0.001)	775 (71.9)	124.075 ** (<0.001)	107.743 ** (<0.001)
	Yes		2813 (14.1)		2537 (13.5)		276 (28.1)		
Work conditions	Quantitative demands	Low	19,833 (86.3)	8120.501 ** (<0.001)	18,982 (86.6)	7926.346 ** (<0.001)	851 (80.2)	230.892 ** (<0.001)	21.540 ** (<0.001)
		High	3322 (13.7)		3121 (13.4)		201 (19.8)		
	Job autonomy	Low	3582 (16.2)	6683.487 ** (<0.001)	3389 (16.1)	6434.683 ** (<0.001)	193 (19.1)	249.770 ** (<0.001)	4.081 * (0.043)
		High	19,528 (83.8)		18,723 (83.9)		859 (80.9)		
	Emotional demands	Low	4457 (19.4)	5444.098 ** (<0.001)	4400 (20.1)	4974.540 ** (<0.001)	57 (4.8)	567.706 ** (<0.001)	98.812 ** (0.001)
		High	18,700 (80.6)		17,705 (79.9)		995 (95.2)		
	Job stress	Low	3963 (16.3)	6902.715 ** (<.001)	3869 (16.6)	6455.187 ** (<0.001)	94 (8.9)	480.929 ** (<0.001)	30.068 ** (<0.001)
		High	19,195 (83.7)		18,237 (83.4)		958 (91.1)		
	Social support from colleagues	Low	971 (4.1)	12,680.737 ** (<0.001)	925 (4.1)	12171.086 ** (<0.001)	46 (4.2)	513.222 ** (<0.001)	0.011 (0.917)
		High	21,077 (95.9)		20,129 (95.9)		948 (95.8)		
	Social support from managers	Low	1403 (5.6)	12,450.037 ** (<0.001)	1323 (5.6)	11,977.056 ** (<0.001)	80 (6.9)	486.269 ** (<0.001)	2.264 (0.132)
		High	20,886 (94.4)		19,952 (94.4)		934 (93.1)		
Presenteeism	No		19,386 (84.1)	7059.339 ** (<0.001)	18,775 (85.3)	7224.053 ** (<0.001)	611 (58.8)	20.398 ** (<0.001)	333.645 ** (<0.001)
	Yes		3778 (15.9)		3337 (14.7)		441 (41.2)		

N = unweighted count; % = weighted %; F = adjusted F; F^a^ = a difference within the group, respectively; F^b^ = a difference between no and yes group by exposure to adverse social behavior; * *p* < 0.05, ** *p* < 0.001.

**Table 2:** Factors related to presenteeism.

			Presenteeism (*n* = 23,164)		
			OR	95% C.I.	*p*
Demographic	Gender (ref: male)	Female	1.606	1.436–1.797	<0.001
	Age (ref: <40)	≥40	1.209	1.081–1.353	0.001
	Education level (ref: ≥college)	≤Middle school	1.416	1.122–1.786	0.003
		High school	1.024	0.903–1.162	0.708
	Income (million won) (ref: ≥5.00)	<1.00	0.848	0.542–1.326	0.470
		1.00–2.99	0.827	0.667–1.025	0.083
		3.00–4.99	1.000	0.820–1.220	0.998
Occupation	Job type (ref: professionals)	Clerks	1.059	0.918–1.222	0.431
		Service and sales workers	0.870	0.742–1.020	0.085
		Blue collar	0.864	0.725–1.028	0.099
	Hours worked per week (ref: ≤40)	41–52	1.330	1.189–1.488	<0.001
		≥53	1.765	1.521–2.048	<0.001
	Company size (ref: <50)	50–249	1.032	0.907–1.174	0.630
		≥250	1.055	0.890–1.249	0.539
	Year of service (ref: ≥10)	<5	0.985	0.858–1.131	0.830
		5–9	1.119	0.980–1.278	0.095
	Work shift (ref: no)	Yes	1.277	1.101–1.428	0.001
	Safety information provided at work (ref: yes)	No	1.133	1.020–1.258	0.019
Adverse social behavior (ref: no)	Yes		2.965	2.452–3.585	<0.001
Discrimination (ref: no)	Yes		1.607	1.412–1.829	<0.001
Work conditions	Quantitative demands (ref: low)	high	1.636	1.440–1.858	<0.001
	Job autonomy (ref: high)	low	1.588	1.407–1.793	<0.001
	Emotional demands (ref: low)	high	1.392	1.204–1.611	<0.001
	Job stress (ref: low)	high	1.543	1.315–1.812	<0.001
	Social support from colleagues (ref: high)	low	0.997	0.767–1.296	0.982
	Social support from managers (ref: high)	low	1.015	0.814–1.266	0.897

OR = odds ratio; CI = confidence interval; ref. = reference.

**Table 3:** Presenteeism among workers exposed to adverse social behavior.

			Presenteeism (*n* = 1052)		
			OR	95% C.I.	*p*
Demographic	Gender (ref: male)	Female	1.147	0.768–1.715	0.502
	Age (ref: <40)	≥40	1.095	0.723–1.657	0.668
	Education level (ref: ≥college)	≤Middle school	0.997	0.408–2.434	0.994
		High school	1.438	0.939–2.203	0.095
	Income (million won) (ref: ≥5.00)	<1.00	0.868	0.177–4.255	0.861
		1.00–2.99	1.143	0.442–2.956	0.783
		3.00–4.99	1.512	0.588–2.892	0.391
Occupation	Job type (ref: professionals)	Clerks	1.346	0.708–2.557	0.365
		Service and sales workers	1.006	0.572–1.769	0.984
		Blue collar	0.974	0.466–2.032	0.943
	Hours worked per week (ref: ≤40)	41–52	1.194	0.781–1.827	0.412
		≥53	1.117	0.683–1.828	0.660
	Company size (ref: <50)	50–249	1.159	0.715–1.880	0.549
		≥250	1.080	0.599–1.948	0.798
	Year of Service (ref: ≥10)	<5	0.807	0.478–1.362	0.422
		5–9	0.950	0.567–1.592	0.847
	Work shift (ref: no)	Yes	1.000	0.664–1.505	0.999
	Safety information provided at work (ref: yes)	No	0.804	0.556–1.164	0.249
Discrimination (ref: no)	Yes		0.956	0.642–1.424	0.824
Work conditions	Quantitative demands (ref: low)	high	1.313	0.861–2.002	0.206
	Job autonomy (ref: high)	low	1.687	1.102–2.580	0.016
	Emotional demands (ref: low)	high	1.437	0.652–3.168	0.369
	Job stress (ref: low)	high	1.145	0.605–2.167	0.677
	Social support from colleagues (ref: high)	low	0.591	0.235–1.487	0.264
	Social support from managers (ref: high)	low	1.301	0.642–2.636	0.465

OR = odds ratio; CI = confidence interval; ref. = reference.

**Table 4:** Presenteeism among workers none exposed to adverse social behavior.

			Presenteeism (*n* = 22,112)		
			OR	95% C.I.	*p*
Demographic	Gender (ref: male)	Female	1.632	1.451–1.835	<0.001
	Age (ref: <40)	≥40	1.217	1.082–1.368	0.001
	Education level (ref: ≥college)	≤Middle school	1.469	1.160–1.861	0.001
		High school	0.999	0.876–1.140	0.993
	Income (million won) (ref: ≥5.00)	<1.00	0.855	0.539–1.356	0.506
		1.00–2.99	0.811	0.651–1.011	0.063
		3.00–4.99	0.977	0.798–1.196	0.821
Occupation	Job type (ref: professionals)	Clerks	1.035	0.893–1.199	0.647
		Service and sales workers	0.845	0.715–0.998	0.047
		Blue collar	0.862	0.712–1.020	0.081
	Hours worked per week (ref: ≤40)	41–52	1.339	1.192–1.504	<0.001
		≥53	1.825	1.562–2.133	<0.001
	Company size (ref: <50)	50–249	1.028	0.899–1.176	0.684
		≥250	1.058	0.886–1.262	0.534
	Year of Service (ref: ≥10)	<5	1.002	0.869–1.157	0.975
		5–9	1.134	0.987–1.301	0.075
	Work shift (ref: no)	Yes	1.329	1.134–1.556	<0.001
	Safety information provided at work (ref: yes)	No	1.167	1.047–1.301	0.005
Discrimination (ref: no)	Yes		1.711	1.496–1.957	<0.001
Work conditions	Quantitative demands (ref: low)	high	1.670	1.463–1.906	<0.001
	Job autonomy (ref: high)	low	1.573	1.385–1.787	<0.001
	Emotional demands (ref: low)	high	1.384	1.193–1.606	<0.001
	Job stress (ref: low)	high	1.571	1.329–1.857	<0.001
	Social support from colleagues (ref: high)	low	1.054	0.805–1.381	0.701
	Social support from managers (ref: high)	low	0.984	0.779–1.243	0.891

OR = odds ratio; CI = confidence interval; ref. = reference.
